# Analysis of Several Physical Phenomena Measured on the Metallic Materials Cut by Abrasive Water Jets (AWJ)

**DOI:** 10.3390/ma15217423

**Published:** 2022-10-22

**Authors:** Jakub Gřunděl, Libor M. Hlaváč, Petr Pětroš, Lucie Gembalová

**Affiliations:** Department of Physics, Faculty of Electrical Engineering and Computer Science, VSB–Technical University of Ostrava, 17. listopadu 2172/15, Ostrava-Poruba, 70800 Ostrava, Czech Republic

**Keywords:** abrasive water jet, forces, vibrations, cutting, normal force, tangential force, traverse speed

## Abstract

Cutting using an abrasive water jet is a complex process involving several physical phenomena. This research studies some of them, mostly the influence of selected variables on the measured forces and vibrations. The traverse speed represents one of the key parameters when cutting using the AWJ. In the presented research, a set of experiments was performed on twelve different metal samples, while the force sensor measured the exerted forces and accelerometers measured the vibrations. Ten different types of steel samples of the same dimensions were cut applying five different traverse speeds. The data obtained during these measurements show that an increase in the traverse speed leads to an increase in the measured forces and vibrations. An analogous experiment performed on bronze and duralumin samples of the same dimensions, having applied higher speeds to compensate for the difference in the material structure and properties, completes the presented data. The most important results of the research are that exerted forces in the *z*-axis are higher than those in the *x*-axis, whereas measured vibrations are higher in the *x*-axis. According to our research, the elemental structure, especially the carbide formation, affects the measured forces and vibrations substantially.

## 1. Introduction

Although quite extensive research has been carried out to find the ways to use the AWJ for other machining operations than cutting, such as milling [1], turning [2], piercing [3], grinding [4] and polishing [5], cutting remains its most widely used application. The main advantage of the AWJ machining is its ability to cut through almost each type of material of any given thickness if the jet energy is sufficient. The AWJ machining has been around for approximately 40 years, whereas no complex model enabling machining outcome predictions for all types of materials is available. Both theoretical and regression models have been published describing the behaviour of some specific groups of materials and AWJ parameters. If we consider the publications devoted to the action of the abrasive water jets, which deal with the action of jets on different types of materials (e.g., [6,7]), we can distinguish two different groups of materials based on their properties and, therefore, two basic types of cutting models. One type of model is focused on ductile materials, namely aluminium, copper, or mild steel. One of the basic studies concerning this type of material is presented in [6], where the authors researched the depth of the cut achieved on ductile materials using the AWJ. The second type of model focuses on brittle materials, namely ceramics, glass, or rocks. In publication [7], the authors aimed to develop a model describing the depth of the cut in brittle materials. Various regression models can provide more precise predictions compared to theoretical models, if the parameters used for machining are similar to those used when creating the models. However, when different parameters are applied, predictions gained from a regression model and experimental results might start to differ substantially [8].

The models mentioned above allow users of the AWJ cutting to predict the outcome before the process begins. Throughout the world, there are ongoing studies investigating the possibility of monitoring the process, while it is happening, to ensure that the result is satisfactory. Due to the nature of the AWJ, irregularities in the jet flow, or inhomogeneities of the used material, are not uncommon. Those may manifest themselves as imperfections on cut materials. Monitoring the process is one of the fundamental setbacks of the research. While other machine tools do not disrupt electronic or optical devices used for the process monitoring, the AWJ does, because of the inevitable presence of water and abrasive particles in the environment. That is why some less common monitoring methods are necessary. One branch of research focuses on studying the vibrations of the cutting head used with the help of accelerometers and tries to find ties with the quality of the machining process in relation to the abrasive mass flow [9] and the traverse speed [10,11]. Similarly, some research focuses on the vibrations of the cut material to monitor the cutting process [12], specifically on determining the metrology and topography of the surface [13] or its roughness [14,15]. Other studies aim at researching the relation between vibrations and changing parameters such as the abrasive mass flow rate or the traverse speed [16,17] or the capability of the vibration measurement and analysis to indicate the wear progression of the focusing tube [18]. Another work focuses on the vibrations of the focusing tube influencing the AWJ cutting capability [19].

Different research branches aim toward the acoustic emissions measurement and determining the machining quality based on those emissions [12]. However, due to the amount of information contained within the signals from the acoustic emissions and vibrations, more than 25 percent of the information may be interpreted incorrectly. Mikler stated this in [20], mentioning at the same time that common signal descriptors such as mean value, RMS, or standard deviation do not allow discovering over- or under- abrasion.

The problems mentioned in [20] arise from the dependence of the measuring results on the proportions of the used machine, its stiffness and the size or shape of the machined material. Therefore, some researchers focused on finding and monitoring parameters that depend only on the cutting process. Most of these studies study the forces acting on the machined material. The first study of forces exerted by the AWJ was published in 1989. It was focused on the measured forces concerning the sapphire orifice diameter, the stand-off distance, the abrasive flow rate and the abrasive size [21]. Throughout the years, the measurement of the acting forces has been used to determine the different parameters. For example, in publications [22,23], the authors study whether exerted forces allow the jet diameter calculation for the pure water jet and the AWJ. Study [24] uses exerted forces to investigate the pulsation of the water jet. Nevertheless, studies on the force of the AWJ impact are very rare, because they need a special device for force measurements and the results are quite difficult to interpret.

**Figure 1:**
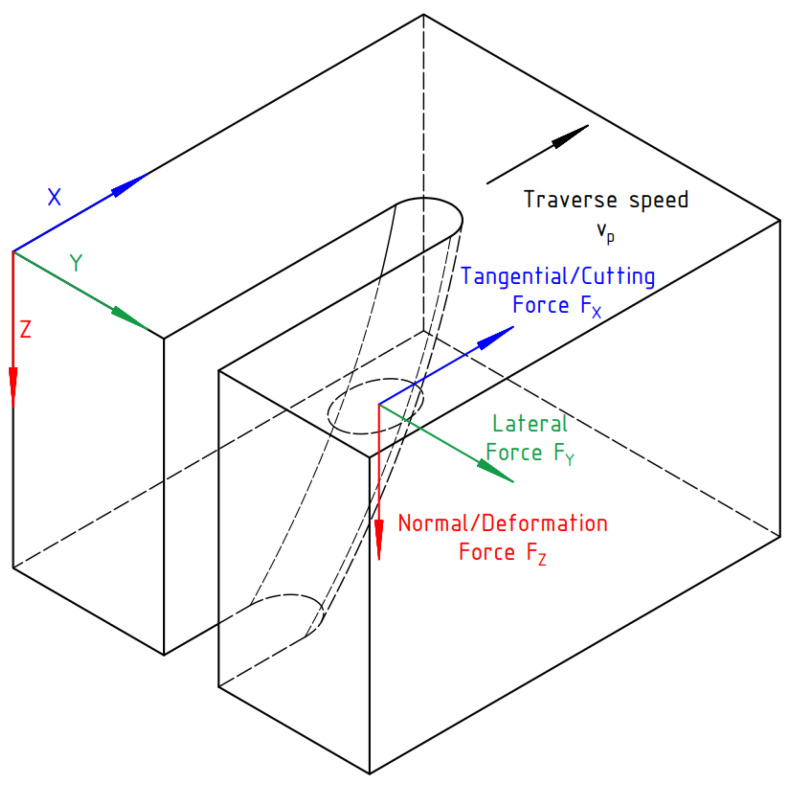
Direction of the exerted forces.

When talking about exerted forces, we recognize three components. Those components denoted in the Cartesian system are forces in x-, y-, and z-axes. Force in the *x*-axis, called the tangential force (TF), acts in the direction of the traversal speed. Force in the *y*-axis, called the lateral force (LF), acts in the same plane perpendicularly to the tangential force. Force in the *z*-axis, called the normal force (NF), usually acts in the direction of the flow of the water jet and it is perpendicular to both the tangential and the lateral force. The tangential force should coincide with the cutting force (CF). The lateral force values usually vary around 50–100 times lower than those of the tangential force [25]. The normal force corresponds with the deformation force (DF). The directions of the exerted forces are sketched in Figure 1.

To measure all the mentioned forces, one must use a particular type of sensor, which measures exerted forces separately for each axis. One specific version of such a force sensor was designed at the VSB–Technical University of Ostrava in 2011 and patented one year later [26]. From this year on, the sensor has been used for a variety of experiments, including the measuring of the exerted forces during the AWJ cutting process. Moreover, 2019 marked the year in which the first reports about the measurements performed using the sensor were presented. Since then, some articles about the results obtained using the sensor, including articles discussing the influence of the material structure on the measured forces [27] and articles researching the possibility of using the force measurement to find the cutting system malfunctions [28] and/or prediction of the machining quality [25], were published.

In the last decade, there has been a significant increase in interest in the use of an abrasive water jet for the micromachining [29,30]. These applications are accompanied by a number of technical difficulties that need to be resolved [31,32]. These problems result in the new challenges that await scientists in this area [33,34]. One of the main problems concerns the sucking of the abrasive material in the very fine fraction (typically, at least MESH 200 and higher), because the abrasive material tends to clog the feed tubes. In order to quickly identify the problems that can affect the quality of the product and the progress of the machining process, it is good to develop the online monitoring and control for the AWJ micro-machines operation. The advantage is that the workpieces are small in size, and therefore it is possible to construct a support device for the workpieces, similar to the force sensor mentioned in this article, which will allow continuous measurement of the forces and possibly also the vibrations. This should help with the monitoring and control of the machining progress so that it is as high-quality and economical as possible.

## 2. Materials and Experimental Setup

The abrasive water jet (AWJ) is generated in two ways. The field machines usually use the so-called slurry jet. This is generated by pressurising of the mixture of water with the abrasive material through the nozzle with the diameter of the millimetre order. The pumps used for generation of this jet are often the direct driven plunger pumps with a rather high flow rate but lower pressures (up to approximately 300 MPa). The machines located in halls, the stable ones, operate usually with pumps multiplying the oil pressure up to the high water-pressure by the piston area ratio (pressures are typically between 250 MPa and 650 MPa). The high-pressure water flows through the water nozzle, often called the orifice, due to its construction. The resulting water jet flows through the chamber, called the mixing chamber, with one or more side inlets for air and the abrasive material. The negative pressure produced by the passing water jet causes a suction of air and abrasive material being poured into the flowing air from the reservoir under its own weight. The mixture of the water, air and abrasive material then flows through the focusing tube that reduces the divergence of the resulting abrasive water jet to the acceptable value.

A series of experiments was performed, upon which this research is based on. The aim of these experiments was to measure simultaneously the exerted forces and vibrations during the AWJ cutting for multiple materials and for five different traverse speeds (TS). The used materials are listed in Table 1.

**Figure 2:**
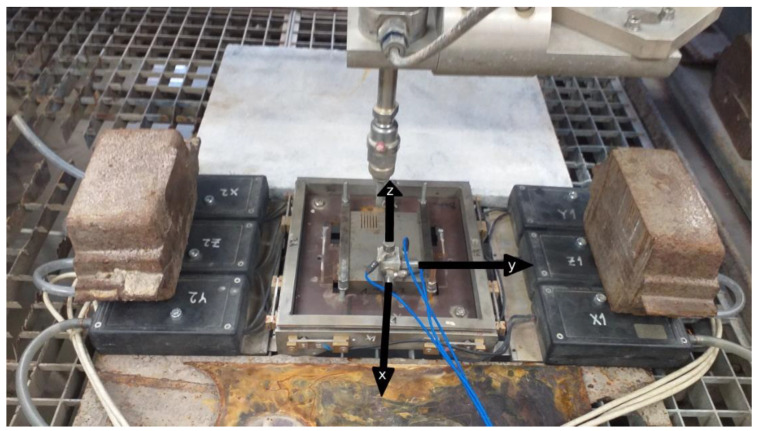
Experimental setup.

The selected materials were machined to have the same dimensions: 90 × 90 × 20 mm. A round 6 mm diameter hole was drilled into them. This hole was used to screw the holder with the accelerometers to the sample for measuring its vibrations. Three 352C33 accelerometers made by PCB Piezotronics (Depew, NY, USA) were used to measure vibrations in the three axes of the Cartesian system. To measure forces in the identical axes, the patented force sensor [26] was used. The sample was tightly attached to a metal plate that was then inserted into the sensor frame. The used setup and axes can be seen in Figure 2.

Both the accelerometers and the force sensor were connected to the A/D transducer PXIe-1071 (National Instruments, Austin, TX, USA). Using NI PXIe-4492 (National Instruments, Austin, TX, USA) for the accelerometers and NI PXIe-6363 (National Instruments, Austin, TX, USA) for the force sensor deformation elements, the signals were transferred to the TravelMate P215-53 laptop (Acer, San Jose, CA, USA) using PXIe-8301 card (National Instruments, Austin, TX, USA). All signals were recorded using the program Signal Express from the National Instruments (Austin, TX, USA). Afterwards, the data were processed using the program LabVIEW (National Instruments, Austin, TX, USA) to receive the average exerted forces and vibrations of the sample from each experiment. Traverse speeds (TS) used during experiments are listed in Table 2.

**Figure 3:**
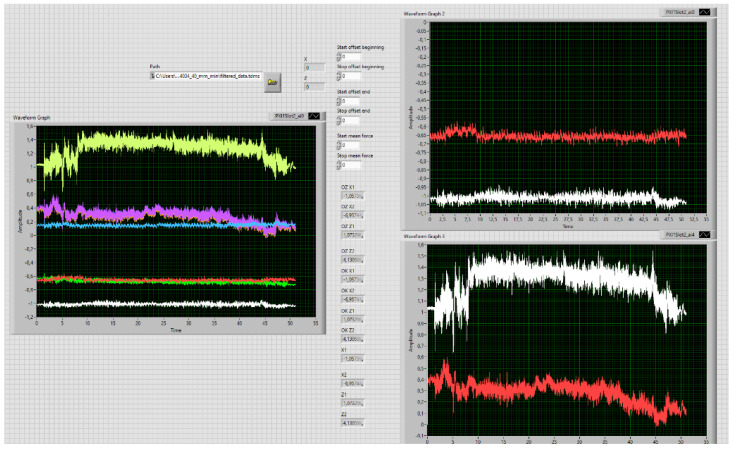
Front panel of the LabVIEW program processing the measured data for steel 1.4034 for the 40 mm/min traverse speed.

The front panel of the program prepared in LabVIEW for processing of data is presented in Figure 3.

Later on, the traverse speeds are labelled as A, B, C, D, E, where A is the lowest speed and E is the highest one, to allow comparison of these speeds for different materials in one table that is more illustrative.

Experiments were performed at the VSB—Technical University of Ostrava using high pressure pump PTV 19/60 on the Flow HSQ 5X basis and the cutting table X-Y CNC WJ1020-1Z-EKO. During all experiments, the identical experimental factors and settings were used. Those are summarized in Table 3.

## 3. Results

The measured average forces and vibrations were saved and analysed in the program Microsoft Excel. To make a full use of the measured data, the multiple theorems were put to the test. The theorems are based on the theoretical descriptions prepared by Hlaváč [37] and they use of the ratio of the pre-set traverse speed and the limit traverse speed for a specific material and the respective AWJ settings.

**Theorem** **1.** *Exerted force in the x-axis is always lower than exerted force in the z-axis. Their ratio is closely dependent on the traverse speed ratio*.

In the previous works, a study of the relation between the ratio of the exerted forces (traverse-to-normal force ratio—TNR) and the traverse speed ratio has been carried out. In [25], an influence of the sample thickness on the relation was studied, while the study presented in [27] was focused on the proof that the behaviour of the ratio is not dependent on the metal composition, strength and density. This was achieved by using the material with the identical elemental composition but different strength characteristics (due to the different heat treatment). The contemporary research is focused on studying the effect of different metal samples with the identical dimensions on the forces ratio regarding the traverse speed ratio.

While other studies aimed at using the corresponding relative traverse speeds so that the results are comparable, the different relative traverse speed is used for all the materials in this research, so that universality of the theorem is reached.

The limit traverse speeds were calculated from equations derived and presented by Hlaváč [37]. The resulting limit speeds were rounded up to tens of millimetres per second, because the regularly used speeds during experiments are dividable by ten. Measured results are listed in Table 4.

(1) $$v_{P\lim}\;=\;{\left[\frac{C_{A}\;S_{p}\;\pi \;d_{o}\;\sqrt{2\rho_{j}p_{j}^{3}\;e^{-5\xi_{j}L}}\;\left(1\;-\;\alpha_{e}^{2}\right)}{8\;H\;\left(p_{j}\rho_{m}\alpha_{e}^{2}\;e^{-2\xi_{j}L}\;+\;\sigma_{m}\rho_{j}\right)}\right]}^{\frac{2}{3}}\;-\;v_{P\min}$$

(2) $$\alpha_{e}=1-\frac{\sqrt{{2p}_{j}^{3}}\;HVt_{i}}{8\sqrt{\rho_{j}}\;\sigma_{m}a_{m}}$$

Nomenclature (basic SI units where available):

$v_{Plim}$

the limit traverse speed;

$C_{A}$

the coefficient modifying abrasive water jet performance according to the changing content of abrasive material below “saturation level” (above this level, the jet performance increases no more and $C_{A}=1$);

$S_{P}$

ratio between the quantity of non-damaged grains (i.e., not containing defects) and the total quantity of grains in the supplied abrasive material;

$d_{o}$

diameter of the water nozzle (orifice);

$\rho_{j}$

density of the abrasive jet (conversion to homogeneous liquid);

$p_{j}$

pressure obtained from Bernoulli’s equation for liquid with density and velocity of the abrasive jet;

$\xi_{j}$

attenuation coefficient of the abrasive jet in the environment between the focusing tube outlet and the material surface;

L

stand-off distance (distance between exit of the focusing tube and material surface);

$\alpha_{e}$

coefficient of the abrasive water jet velocity loss in the interaction with material (experimentally determined);

H

material thickness;

$\rho_{m}$

density of material being machined;

$\sigma_{m}$

strength of material being machined;

$v_{Pmin}$

minimum limit traverse speed of cutting—correction for the traverse speed zero (usually $v_{Pmin}=a_{n}/60$ is used, where $a_{n}$ is the average abrasive particle size after the mixing process inside the mixing head and focusing tube);

HV

material hardness;

$t_{i}$

interaction time;

$a_{m}$

mean size of particles (elements) of material—grains or their chips.

The detailed description of the theoretical base was published in [38].

**Theorem** **2.** *Elemental structure of steel affects exerted forces*.

As mentioned in Theorem 1, the elemental structure of the sample affects the forces that are exerted during the AWJ cutting. It has been studied in [39] that the declination angle of the surface striations is dependent on the structure of the cut material. Certain elements might help to harden the steel, thus increasing the exerted forces, while other elements can reduce the hardening and make the steel more ductile. The elemental structure of the used steel samples can be seen in Table 5, because a comparison of differentness of the composition for bronze and duralumin is redundant.

**Theorem** **3.** *The traverse speed and vibrations of the sample are closely connected. Vibrations increase with increasing traverse speed. The same can be said about the traverse speed and exerted forces*.

When the AWJ cuts through the sample, its kinetic energy is transferred to the sample. Lower speeds cause higher losses of the applied energy because only a fraction of the jet actually hits the sample. Using higher speeds should result in higher values of the measured vibrations and higher values of the exerted forces.

**Theorem** **4.** *Vibrations are dependent on the elemental composition of the used steel*.

As with the forces, the measured vibrations should be influenced by the type of the steel used for the sample, specifically by its elemental composition.

**Theorem** **5.** *Vibrations in the z-axis are lower than those in the x-axis*.

While the exerted forces in the *z*-axis are higher than the ones in the *x*-axis, this theorem claims that vibrations have the opposite behaviour. Due to the continuous effect of the gravity, the vibrations in the *z*-axis are dampened, while those in the *x*-axis are amplified by the flow fluctuation due to the curvature inside the kerf.

## 4. Discussion

Due to the nature of the performed experiments, there are many factors influencing the results of the measured forces. Those factors are connected either to the samples, the measuring devices or the environment, in which the experiments are carried out.

For the samples, those factors include the dimensions of the sample, the homogeneity of the sample, the fixture of the sample and its position on the measuring device. For the force sensor, it can be its hysteresis and stiffness. For the accelerometers, the accuracy of their positioning in the axes is crucial; otherwise, the vibrations may be projected to two or three axes without a definite ratio of their values.

Environmental factors might include the positioning and the weight of the elements used to hold the force sensor in its position on the cutting table, the height of the water level in the attenuating vessel, the type and quality of the grid used to hold the force sensor and the positioning of the sensor on this grid.

These factors are hard to replicate if they are changed. For the performed experiments, the factors remained identical, or if they changed, their change was insignificant. Therefore, performing the repeated experiments might bring different results, but the qualitative behaviour should remain the same.

### 4.1. Discussion of Theorem 1

The authors of [25] already proposed and tested the theorem regarding the ratio of the tangential to the normal force and the ratio of the used traverse speed to the limit traverse speed. According to this theorem, the ratio of forces increases with increasing the speed. When using thicker samples, the respective ratio can stay high and does not decrease, whereas when thinner samples are used, the ratio decreases as the traverse speed approaches the limit traverse speed. The results are presented in Figure. 6 in the cited article [25].

When the traverse speed increases, more of the kinetic energy of the AWJ is transferred to the cut material, resulting in the increase in the normal force. This also leads to an increase in the tangential force, because of the lagging and the flow curvature that is connected to it. When using thinner samples, the normal force can increase over the limit seen in the thicker samples, resulting in a decrease in the TNR.

**Figure 4:**
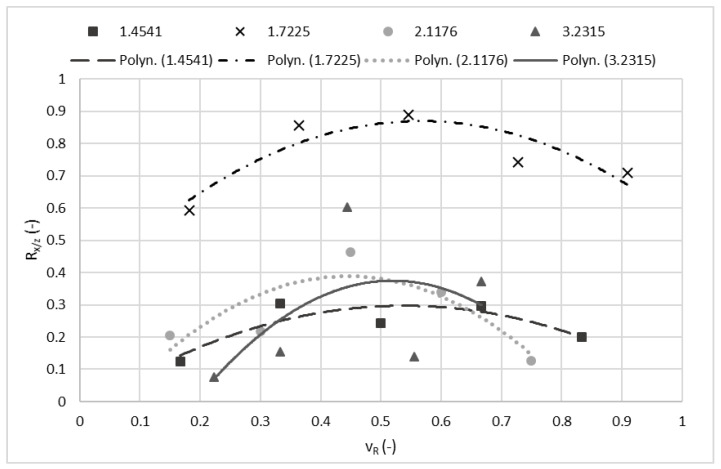
Ratio (R_x/z_) of the *x*-axis forces to the *z*-axis forces in relation to the relative traverse speed.

To test the theorem, four samples—steel 1.4541; steel 1.7225; bronze 2.1176; duralumin 3.2315—were selected. The values of the measured forces can be seen in Table 6, whereas their ratio is presented in Table 7. Respective data are plotted on a graph in Figure 4 and interleaved with a second order polynomial curve. The uncertainty of the measurement was up to 5%.

It can be seen that the highest number of the measured values appears close to the regression curve, which means that the results are fairly accurate. The measurement performed on the duralumin has a higher uncertainty and, therefore, more experiments should be performed in the future to figure out the cause.

**Proof** **of** **Theorem** **1.** Results presented in Table 7 and Figure 4 confirm that never during the experiments was the average tangential force exerted higher than the respective normal force. This behaviour of the ratios matches the behaviour predicted in Theorem 1 and in the article [25].

### 4.2. Discussion of Theorem 2

**Figure 5:**
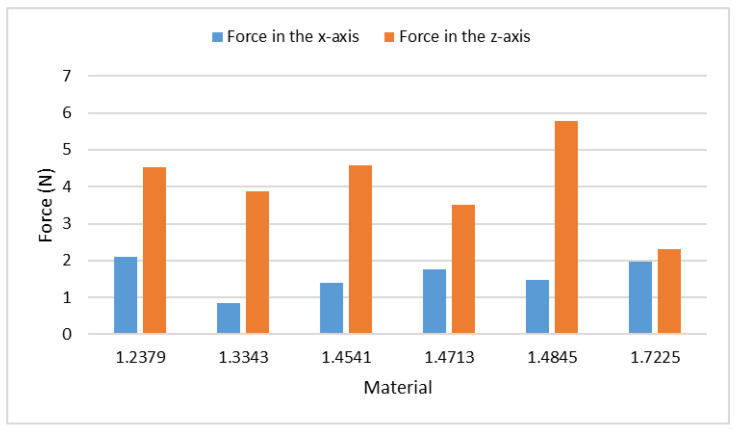
Measured forces at the traverse speed 40 mm/min on selected steels.

The elemental composition of studied steels (see Table 5) indicates that the most significant difference distinguishing the selected steels is their content of chromium. The articles aimed at the wear of the cast iron with a high amount of chromium state that the chromium increases the carbide formation [40,41]. The higher percentage of chromium also increases the brittleness and abrasive resistance [42,43]. Opposite to it, the higher amount of nickel increases the steel toughness and plasticity [44,45]. Because carbides are made up of carbon and other metallic and semi-metallic elements, the amount of carbon in steels needs to be also considered. Higher number of carbides usually strengthen the steel, which means that higher force must be applied to successfully cut the sample. Another reason why carbides increase forces is that carbides are often harder than the used abrasive material and the whole grain of carbide must be removed during impact. Therefore, the greater carbides increase vibrations and forces, while the smaller ones do not. Measured forces can be seen in Table 8, and the bar graph presented in Figure 5 shows their plots. The experiments, performed applying the identical speed (40 mm/min), have been selected for this comparison.

**Figure 6:**
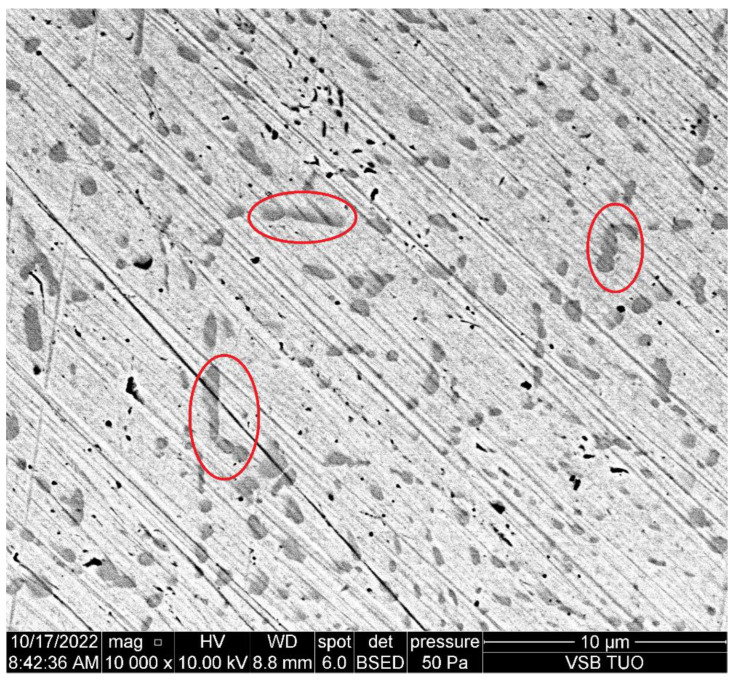
Carbides in the steel 1.4034 (picture from the SEM microscope). Red circles show the largest carbides in the picture.

Restricting Table 5 to the tested steels (Table 8, Figure 5), Table 9 presents only the important elements—carbon and chromium. It can be seen that the steel 1.4845 with the highest percentage of chromium also has the highest force in the *z*-axis. This is caused by a higher number of small carbides influencing the deformation mode of the material removal. On the other hand, the steel 1.7225 with the lowest percentage of chromium inside shows the lowest *z*-axis force. The similarity in the measured forces on steels 1.2379 and 1.4541 can be caused by a compensation of the higher percentage of chromium by the lower percentage of carbon and vice versa. Forces in the *x*-axis seem to be less dependent on the structure, and more research needs to be done in this area. Figure 6 shows the carbides in the 1.4034 steel.

**Proof** **of** **Theorem** **2.** It has been proven that the elemental structure has impact mainly on the measured forces in the *z*-axis; the results in the *x*-axis seem to be less affected. The number of carbides in the steel sample helps to determine the level of the exerted force, whereas knowledge of the concentration of certain elements allows knowing approximately the number of carbides in the sample.

### 4.3. Discussion of Theorem 3

The traverse speed is one of two parameters that directly impacts the quality of the cut, the other one being the pump pressure. All other parameters are in some form linked to the various parameters. For example, the abrasive mass flow rate is dependent on the used pump pressure and the water flow rate. The applied water nozzle and the focusing tube diameters affect quality in relation to one another—the changing of one without the other might result in both an increase or a decrease in the quality at all times. Simultaneously, the water nozzle diameter and the focusing tube diameter determine an appropriate mean abrasive grain size that can be efficiently sucked into the mixing chamber, as well as the resulting mean particle size in the mixture outflowing from the focusing tube.

**Figure 7:**
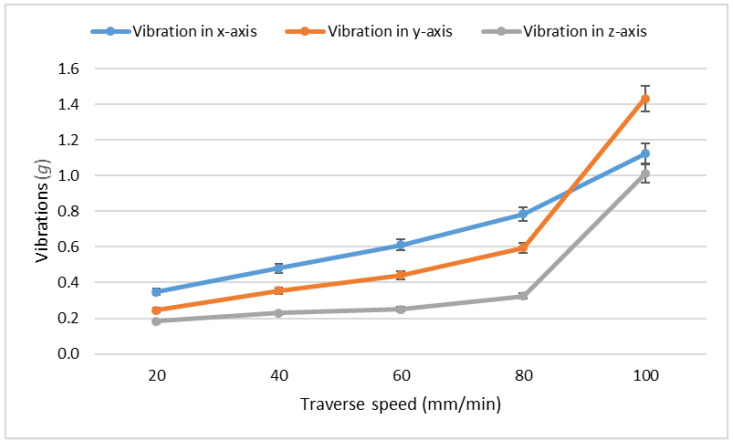
Measured vibrations in relation to the traverse speed on the steel 1.2379.

**Figure 8:**
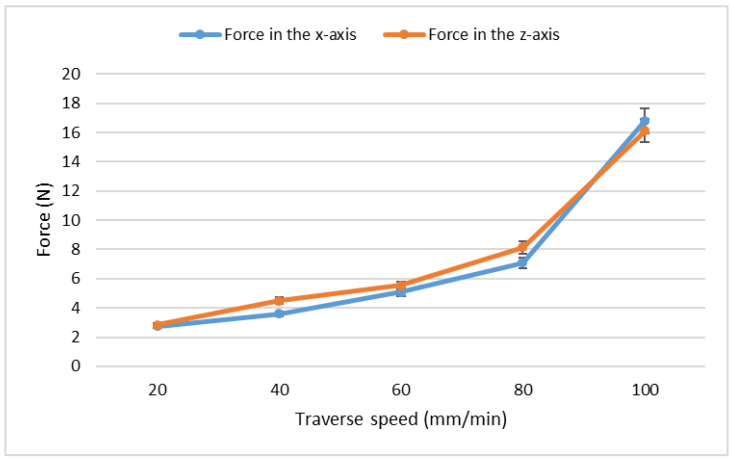
Measured forces in relation to the traverse speed on the steel 1.2379.

The question remains whether the traverse speed affects the vibrations and forces in the same way as it does with the quality of the cut. Experiments were conducted on the steel 1.2379 and the measured vibrations can be seen in Table 10, while the measured forces are presented in Table 11. Data from Table 10 were plotted in Figure 7 and data from Table 11 were plotted in Figure 8.

From the results in both the tables and the figures, it can be seen that the vibrations and the exerted forces really increase with the increasing traverse speed. The rapid growth of values at the speed 100 mm/min might be attributed to the slight hitting of the support grid, on which the force sensor was situated, by the residual abrasive water jet. The reflected jet impacts the support with the steel sample, thus increasing both the vibrations and the measured forces. However, the functional dependency of the forces and vibrations on the traverse speeds strongly resembles the dependencies for the declination angle presented in the article [37].

**Proof** **of** **Theorem** **3.** Data from the presented experiments prove that the theorem is correct at least for the studied steel sample. Repeating this experiment might result in slight deviations but both the vibrations and the forces should increase with the increasing traverse speed.

### 4.4. Discussion of Theorem 4

Just as the exerted forces are dependent on the elemental structure, the vibrations should be also somehow affected by the concentration of certain elements in the structure. Findings from Theorem 2 are considered, and attention is paid especially to chromium and carbon.

**Figure 9:**
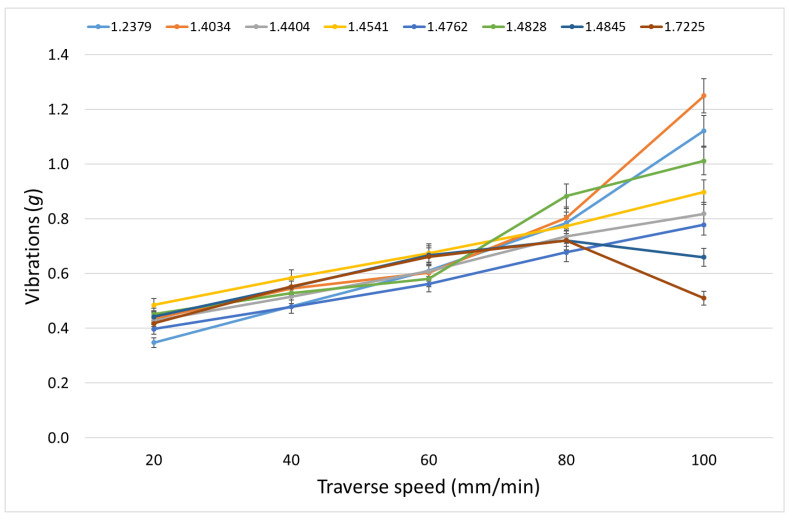
Vibrations in the *x*-axis on selected steels for tested traverse speeds.

**Figure 10:**
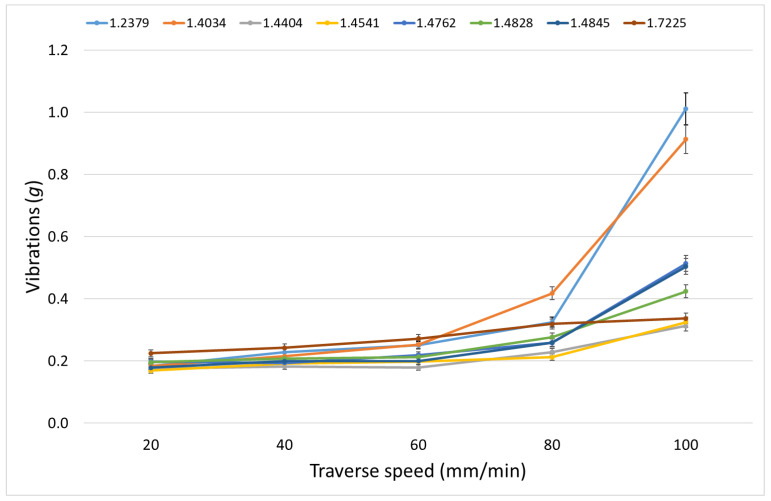
Vibrations in the *z*-axis on selected steels for tested traverse speeds.

Table 12 shows the measured vibrations in the x-axis, and Table 13 shows those in the z-axis. In Figure 9, vibrations in the *x*-axis are plotted, and Figure 10 presents data from Table 13.

Table 14 shows the reduced elemental concentration of selected steels, highlighting the percentage of carbon and chromium.

A higher percentage of carbon and chromium increases the number of chromium carbides, which can cause a higher resistance of steel against the AWJ penetration at higher traverse speeds in the case of the cutting and deformation mode. This is particularly noticeable on 1.2379 and 1.4034 steels. On the other hand, if the percentage of chromium is low, the formation of carbides is limited, and the result can be a decrease in vibrations at higher traverse speeds (steel 1.7225). The low percentage of carbon limits the formation of carbides even with a higher percentage of chromium, and the resulting vibrations in the x-axis then increase practically linearly with the increasing traverse speed, which is noticeable mainly on steels 1.4404, 1.4541, 1.4762 and 1.4828. Vibrations in the z-axis follow the behaviour of the relation between the declination angle and the traverse speed.

**Proof** **of** **Theorem** **4.** The results presented in Table 12, Table 13 and Table 14 and Figure 9 and Figure 10 prove that vibrations in the x- and the z-axes are affected by the number of chromium carbides in the steels.

### 4.5. Discussion of Theorem 5

**Figure 11:**
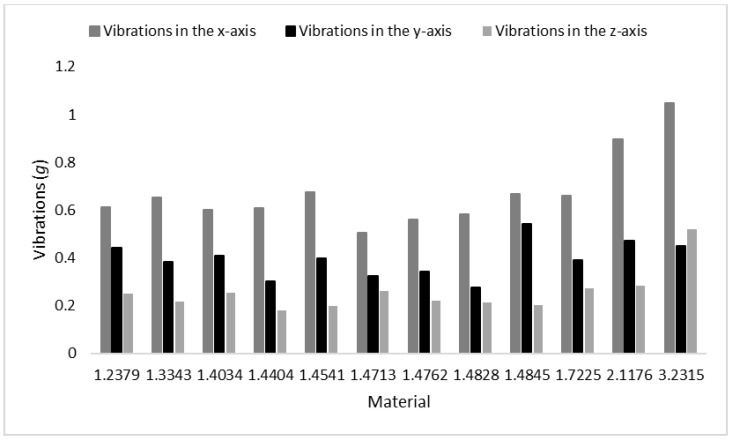
Vibrations in relation to the material type using the speed C.

Vibrations were measured for the different types of steel materials using the identical traverse speed for all of them (60 mm/min) and the higher speeds for the bronze (90 mm/min) and the duralumin (160 mm/min). The traverse speeds were selected so that the expected declination angle [37] was similar to the one on steels. Measured results are listed in Table 15 and plotted in Figure 11.

As it is evident in Figure 11, vibrations in the *x*-axis are always the highest ones, while vibrations in the *z*-axis are almost always the lowest ones with the exception of the duralumin. Low vibrations in the *z*-axis can be attributed to the gravitational force acting as a damper of the sample movement. The duralumin sample has lower weight, so the gravitational force is lower, and vibrations are not so dampened. Vibrations in the *y*-axis are similar for all samples. The material effect in this axis seems to be insignificant. These vibrations are influenced mainly by the instabilities of the pressure and the respective fluctuation of the abrasive flow rate. Vibrations in the *x*-axis are the highest ones because they include the effect of the cutting mode of the material removal and the respective fluctuation of the curved motion of the jet inside the produced kerf. The force relief during the local removal of the material from the kerf head and the re-increase in the force action on the newly formed kerf head are the main sources of fluctuations in the cutting forces.

**Proof** **of** **Theorem** **5.** The theorem that vibrations in the *x*-axis are higher than the ones in the *z*-axis has been proven by the measured data and their discussion.

## 5. Conclusions

For the research presented in this article, the multiple experiments aimed at the physical phenomena present during the AWJ cutting were performed using the force sensor and accelerometers, with the goal of testing several theorems. The conclusions resulting from those experiments are as follows:

1. The measured forces in the *x*-axis are always lower than forces in the *z*-axis;
2. The exerted forces are dependent on the elemental structure of the material;
3. The number of carbides in steels affects the measured forces—a higher concentration of carbides results in higher forces;
4. The concentration of chromium and carbon substantially affect the formation of carbides in steels and, subsequently, their response to the AWJ machining;
5. Increasing the traverse speed increases both the measured forces and the vibrations;
6. The measured vibrations are affected by the elemental structure of machined material;
7. The vibrations are dependent on the number of carbides in steels; a higher number of carbides leads to higher vibrations;
8. The vibrations in the *x*-axis are higher than those in the *z*-axis due to the gravitational force damping the movement in the *z*-axis in used configurations.

The further research will be aimed at some nonhomogeneous materials, namely rocks, glass and composites. One of the future steps is a preparation of the device enabling the monitoring of the micromachining process in real time and possibly even the ability to control it. Nevertheless, the priority in the nearest future is to perform more experiments on a broad scale of materials, not only metals.

## Figures and Tables

**Table 1:** Properties of used materials selected from the material sheets summarized in lexicon [35].

Material	Type	Grade	Density kg/m^3^	Hardness HB Max	Yield Strength MPa
1.2379	Tool steel	X153CrMoV12	7600	250	845
1.3343	High speed tool steel	HS6-5-2C	8200	255	850
1.4034	Stainless steel	X46Cr13	7700	245	780
1.4404	Stainless steel	X2CrNiMo17-12-2	8000	-	595
1.4541	Stainless steel	X6CrNiTi18-10	7900	-	600
1.4713	Heat resisting steel	X10CrAlSi7	7700	192	520
1.4762	Heat resisting steel	X10CrAlSi25	7700	223	620
1.4828	Heat resisting steel	X15CrNiSi20-12	7900	223	650
1.4845	Heat resisting steel	X8CrNi25-21	7900	192	600
1.7225	Alloy special steel	42CrMo4	7850	-	1100
2.1176	Bronze alloy	CuSn10Pb10	8900	65	180
3.2315	Aluminium alloy	AlSi1MgMn	2700	83	295

**Table 2:** Traverse speeds used during experiments.

Used Material	Used Traverse Speeds (mm/min)
1.2379, 1.3343, 1.4034, 1.4404, 1.4541, 1.4713, 1.4762, 1.4828, 1.4845, 1.7225	20, 40, 60, 80, 100
2.1176	30, 60, 90, 120, 150
3.2315	80, 120, 160, 200, 240

**Table 3:** Factors and settings applied in all experiments.

Variable	Value
Pump pressure	380 MPa
Water nozzle diameter	0.25 mm
Focusing tube diameter	0.76 mm
Focusing tube length	76 mm
Abrasive mass flow rate	250 g/min
Abrasive material mean grain size (input)	0.250 mm
Abrasive material mean grain size in jet ^1^	0.024 mm
Abrasive material type	Australian garnet (GMA80)
Stand-off distance	2 mm

^1^ Calculated from the initial factors and settings according to [36].

**Table 4:** Limit traverse speed (LTS) and the traverse speed ratios for selected values of TS.

Material	LTS (mm/min)	TS A	TS B	TS C	TS D	TS E
1.4541	120	0.17	0.33	0.50	0.67	0.83
1.7225	110	0.18	0.36	0.55	0.73	0.91
2.1176	200	0.15	0.30	0.45	0.60	0.75
3.2315	360	0.22	0.33	0.44	0.56	0.67

**Table 5:** Maximal elemental composition (mass percentage) selected for used steels from lexicon of steels [35].

Material	C	Si	Mn	P	S	Cr	Mo	V	Ni	N	Ti	Al	W
1.2379	1.60	0.40	0.45	0.030	0.030	12.0	0.80	1.10	-	-	-	-	-
1.3343	0.94	0.45	0.40	0.030	0.030	4.5	5.20	2.00	-	-	-	-	6.70
1.4034	0.50	1.00	1.00	0.040	0.015	14.5	-	-	-	-	-	-	-
1.4404	0.03	1.00	2.00	0.045	0.015	18.5	2.50	-	13.0	0.11	-	-	-
1.4541	0.08	1.00	2.00	0.045	0.015	19.0	-	-	12.0	-	0.70	-	-
1.4713	0.12	1.00	1.00	0.040	0.015	8.0	-	-	-	-	-	1.00	-
1.4762	0.12	1.40	1.00	0.040	0.015	26.0	-	-	-	-	-	1.70	-
1.4828	0.20	2.50	2.00	0.045	0.015	21.0	-	-	13.0	0.11	-	-	-
1.4845	0.10	1.50	2.00	0.045	0.015	26.0	-	-	22.0	0.11	-	-	-
1.7225	0.45	0.40	0.90	0.035	0.035	1.2	0.30	-	-	-	-	-	-

**Table 6:** Forces in the x- and z-axes in relation to the selected materials and the traverse speeds.

Material	Axis	Force for TS A (N)	Force for TS B (N)	Force for TS C (N)	Force for TS D (N)	Force for TS E (N)
1.4541	x	0.37	1.39	1.28	2.40	1.83
	z	3.00	4.58	5.29	8.10	9.16
1.7225	x	1.51	1.98	4.19	4.37	3.86
	z	2.54	2.31	4.71	5.88	5.43
2.1176	x	0.44	0.77	2.13	1.61	0.89
	z	2.13	3.51	5.06	9.28	7.00
3.2315	x	0.17	0.61	3.61	1.30	3.50
	z	2.26	3.92	5.99	0.14	9.37

**Table 7:** Ratio of x- and z-axes forces (TNR) in relation to selected materials and traverse speeds.

Material	TNR Using TS A	TNR Using TS B	TNR Using TS C	TNR Using TS D	TNR Using TS E
1.4541	0.12	0.30	0.24	0.30	0.20
1.7225	0.59	0.86	0.89	0.74	0.71
2.1176	0.21	0.22	0.46	0.34	0.13
3.2315	0.08	0.15	0.60	0.14	0.37

**Table 8:** Measured forces at the traverse speed 40 mm/min on selected steels.

Material	Force in the *x*-Axis (N)	Force in the *z*-Axis (N)
1.2379	2.10	4.51
1.3343	0.84	3.87
1.4541	1.39	4.58
1.4713	1.75	3.50
1.4845	1.47	5.78
1.7225	1.98	2.31

**Table 9:** Elemental structure selected from Table 5 for steels presented in Table 8 and Figure 5.

Material	C (%)	Cr (%)
1.2379	1.60	12.0
1.3343	0.94	4.5
1.4541	0.08	19.0
1.4713	0.12	8.0
1.4845	0.10	26.0
1.7225	0.45	1.2

**Table 10:** Measured vibrations on the steel 1.2379 in multiples of gravitational acceleration (*g*).

Speed (mm/min)	Vibration in *x*-Axis (*g*)	Vibration in *y*-Axis (*g*)	Vibration in *z*-Axis (*g*)
20	0.35	0.24	0.18
40	0.48	0.35	0.22
60	0.61	0.44	0.25
80	0.78	0.59	0.33
100	1.12	1.43	1.01

**Table 11:** Measured forces on the steel 1.2379 in Newtons.

Speed (mm/min)	Force in the *x*-Axis (N)	Force in the *z*-Axis (N)
20	2.75	2.84
40	3.60	4.50
60	5.08	5.53
80	7.09	8.14
100	16.80	16.12

**Table 12:** Vibrations measured in the *x*-axis in relation to the respective steel and the traverse speed.

Speed (mm/min)	1.2379 (*g*)	1.4034 (*g*)	1.4404 (*g*)	1.4541 (*g*)	1.4762 (*g*)	1.4828 (*g*)	1.4845 (*g*)	1.7225 (*g*)
20	0.35	0.44	0.43	0.49	0.40	0.45	0.44	0.42
40	0.48	0.54	0.52	0.58	0.48	0.53	0.55	0.55
60	0.61	0.60	0.61	0.67	0.56	0.58	0.67	0.66
80	0.78	0.80	0.74	0.77	0.68	0.88	0.72	0.72
100	1.12	1.25	0.82	0.90	0.78	1.01	0.66	0.51

**Table 13:** Vibrations measured in the *z*-axis in relation to the respective steel and the traverse speed.

Speed (mm/min)	1.2379 (*g*)	1.4034 (*g*)	1.4404 (*g*)	1.4541 (*g*)	1.4762 (*g*)	1.4828 (*g*)	1.4845 (*g*)	1.7225 (*g*)
20	0.18	0.18	0.18	0.17	0.20	0.20	0.18	0.22
40	0.23	0.22	0.18	0.19	0.19	0.21	0.20	0.24
60	0.25	0.25	0.18	0.20	0.22	0.21	0.20	0.27
80	0.33	0.42	0.23	0.21	0.26	0.28	0.26	0.32
100	1.01	0.91	0.31	0.32	0.51	0.42	0.50	0.34

**Table 14:** Elemental concentration of carbon and chromium in selected steels.

Material	C (%)	Cr (%)
1.2379	1.60	12.0
1.4034	0.50	14.5
1.4404	0.03	18.5
1.4541	0.08	19.0
1.4762	0.12	26.0
1.4828	0.20	21.0
1.4845	0.10	26.0
1.7225	0.45	1.2

**Table 15:** Vibrations in relation to the material type using speed C.

Material	Vibration in *x*-Axis (*g*)	Vibration in *y*-Axis (*g*)	Vibration in *z*-Axis (*g*)
1.2379	0.61	0.44	0.25
1.3343	0.65	0.38	0.22
1.4034	0.60	0.41	0.25
1.4404	0.61	0.30	0.18
1.4541	0.67	0.40	0.20
1.4713	0.51	0.32	0.26
1.4762	0.56	0.34	0.22
1.4828	0.58	0.28	0.21
1.4845	0.67	0.54	0.20
1.7225	0.66	0.39	0.27
2.1176	0.90	0.47	0.28
3.2315	1.05	0.45	0.52

## Data Availability

Not applicable.

## References

[1] Rabani A., Madariaga J., Bouvier C., Axinte D. (2016). An approach for using iterative learning for controlling the jet penetration depth in abrasive waterjet milling. J. Manuf. Process..

[2] Zohourkari I., Zohoor M., Annoni M. (2014). Investigation of the effects of machining parameters on material removal rate in abrasive waterjet turning. Adv. Mech. Eng..

[3] Schwartzentruber J., Papini M. (2015). Abrasive waterjet micro-piercing of borosilicate glass. J. Mater. Process. Technol..

[4] Liang Z.W., Xie B.H., Liao S.P., Zhou J.H. (2015). Concentration degree prediction of AWJ grinding effectiveness based on turbulence characteristics and the improved ANFIS. Int. J. Adv. Manuf. Technol..

[5] Loc P.H., Shiou F.J., Lin Z.C., Huang Y.M., Chen C.C.A., Chen L.K. (2012). Abrasive water jet polishing on Zr-based bulk metallic glass. Advanced Materials Research.

[6] Paul S., Hoogstrate A.M., van Luttervelt C.A., Kals H.J.J. (1998). Analytical and Experimental Modeling of Abrasive Water Jet Cutting of Ductile Materials. J. Mater. Process. Technol..

[7] Paul S., Hoogstrate A.M., van Luttervelt C.A., Kals H.J.J. (1998). Analytical Modeling of the Total Depth of Cut in Abrasive Water Jet Machining of Polycrystalline Brittle Materials. J. Mater. Process. Technol..

[8] Hlaváč L.M., Krajcarz D., Hlaváčová I.M., Spadło S. (2017). Precision comparison of analytical and statistical-regression models for AWJ cutting. Precis. Eng..

[9] Fabian S., Salokyová Š. (2013). AWJ cutting: The technological head vibrations with different abrasive mass flow rates. Appl. Mech. Mater..

[10] Salokyová Š. (2014). Measurement and analysis of technological head vibrations in hydro-abrasive cutting technology. Acad. J. Manuf. Eng..

[11] Salokyová Š. (2016). Measurement and analysis of mass flow and feed speed impact on technological head vibrations during cutting abrasion resistant steels with abrasive water jet technology. Key Eng. Mater..

[12] Hloch S., Ruggiero A. (2013). Online monitoring and analysis of hydroabrasive cutting by vibration. Adv. Mech. Eng..

[13] Hreha P., Hloch S. (2013). Potential use of vibration for metrology and detection of surface topography created by abrasive waterjet. Int. J. Surf. Sci. Eng..

[14] Hreha P., Radvanska A., Knapcikova L., Królczyk G.M., Legutko S., Królczyk J.B., Hloch S., Monka P. (2015). Roughness parameters calculation by means of on-line vibration monitoring emerging from AWJ interaction with material. Metrol. Meas. Syst..

[15] Monno M., Ravasi C. (2005). The effect of cutting head vibrations on the surfaces generated by waterjet cutting. Int. J. Mach. Tools Manuf..

[16] Prislupčák M., Panda A., Jančík M., Pandová I., Orendáč P., Krenický T. (2014). Diagnostic and Experimental Valuation on Progressive Machining Unit. Applied Mechanics and Materials.

[17] Olejarova S., Krenicky T. (2021). Water Jet Technology: Experimental Verification of the Input Factors Variation Influence on the Generated Vibration Levels and Frequency Spectra. Materials.

[18] Copertaro E., Perotti F., Annoni M. (2021). Operational vibration of a waterjet focuser as means for monitoring its wear progression. Int. J. Adv. Manuf. Technol..

[19] Copertaro C., Perotti F., Castellini P., Chiariotti P., Martarelli M., Annoni M. (2020). Focusing tube operational vibration as a means for monitoring the abrasive waterjet cutting capability. J. Manuf. Processes.

[20] Mikler J. (2014). On use of acoustic emission in monitoring of under and over abrasion during a water jet milling process. J. Mach. Eng..

[21] Li H.Y., Geskin E.S., Chen W.L., Vijay M.M., Savanick G.A. (1989). Investigation of forces exerted by an abrasive water jet on workpiece. Proceedings of the 5th American Water Jet Conference.

[22] Kliuev M., Pude F., Stirnimann J., Wegener K., Klichová D., Sitek L., Hloch S., Valentinčič J. (2021). Measurement of the effective waterjet diameter by means of force signals. Proceedings of the Advances in Water Jetting—Water Jet 2019.

[23] Orbanic H., Junkar M., Bajsic I., Lebar A. (2009). An instrument for measuring abrasive water jet diameter. Int. J. Mach. Tools Manu..

[24] Foldyna J., Sitek L., Švehla B., Švehla T. (2004). Utilization of ultrasound to enhance high-speed water jet effects. Ultrason. Sonochem..

[25] Hlaváč L.M., Annoni M.P.G., Hlaváčová I.M., Arleo F., Viganò F., Štefek A. (2021). Abrasive Waterjet (AWJ) Forces—Potential Indicators of Machining Quality. Materials.

[26] Mádr V., Lupták M., Hlaváč L. (2012). Force Sensor and Method of Force Sensing in the Process of Abrasive Water Jet. Cutting. Patent No..

[27] Hlaváč L.M., Štefek A., Tyč M., Krajcarz D. (2020). Influence of Material Structure on Forces Measured during Abrasive Waterjet (AWJ) Machining. Materials.

[28] Hlaváč L.M., Bańkowski D., Krajcarz D., Štefek A., Tyč M., Młynarczyk P. (2021). Abrasive Waterjet (AWJ) Forces—Indicator of Cutting System Malfunction. Materials.

[29] Shi L.P., Fang Y., Dai Q.W., Huang W., Wang X.L. (2018). Surface texturing on SiC by multiphase jet machining with microdiamond abrasives. Mater. Manuf. Process..

[30] Mehta K.M., Pandey S.K., Shaikh V.A. (2021). Unconventional Machining of ceramic matrix Composites—A review. Mater. Today Proc..

[31] Hou R.G., Wang T., Lv Z., Liu Y.Y. (2018). Experimental Study of the Ultrasonic Vibration-Assisted Abrasive Waterjet Micromachining the Quartz Glass. Adv. Mater. Sci. Eng..

[32] Singh D., Shukla R. (2020). Multi-objective optimization of selected non-traditional machining processes using NSGA-II. Decis. Sci. Lett..

[33] Debnath S., Kunar S., Anasane S.S., Bhattacharyya B., Kibria G., Bhattacharyya B., Davim J.P. (2017). Non-traditional Micromachining Processes: Opportunities and Challenges. Non-Traditional Micromachining Processes: Fundamentals and Applications.

[34] Melentiev R., Fang F.Z. (2018). Recent advances and challenges of abrasive jet machining. CIRP J. Manuf. Sci. Technol..

[35] Fürbacher I., Macek K., Steidl J. (1998). Lexikon Technických Materiálů se Zahraničními Ekvivalenty (Lexicon of Technical Materials with Foreign Equivalents).

[36] Hlaváč L.M., Martinec P., Louis H. (1998). Almandine garnets as abrasive material in high-energy waterjet—Physical modelling of interaction, experiment and prediction. Proceedings of the 14th International Conference on Jetting Technology.

[37] Hlaváč L.M. (2009). Investigation of the abrasive water jet trajectory curvature inside the kerf. J. Mater. Process. Technol..

[38] Hlaváč L.M. (2021). Revised Model of Abrasive Water Jet Cutting for Industrial Use. Materials.

[39] Strnadel B., Hlaváč L.M., Gembalová L. (2013). Effect of steel structure on the declination angle in AWJ cutting. Int. J. Mach. Tools Manuf..

[40] Pokusová M., Brúsilová A., Šooš L., Berta I. (2016). Abrasion Wear Behavior of High-chromium Cast Iron. Arch. Foundry Eng..

[41] Atabaki M.M., Jafari S., Abdollah-pour H. (2012). Abrasive Wear Behavior of High Chromium Cast Iron and Hadfield Steel—A Comparison. J. Iron Steel Res..

[42] Titov V.I., Tarasenko L.V., Utkina A.N. (2017). Effect of alloying elements on the composition of carbide phases and mechanical properties of the matrix of high-carbon chromium–vanadium steel. Phys. Met. Metallogr..

[43] Kagawa A., Kawashima S., Ohta Y. (1992). Wear Properties of (Fe, Cr)7C3 Carbide Bulk Alloys. Mater. Trans. Jim.

[44] Chakraborty G., Kumar N., Das C.R., Albert S.K., Bhaduri A.K., Dash S., Tyagi A.K. (2014). Study on microstructure and wear properties of different nickel base hardfacing alloys deposited on austenitic stainless steel. Surf. Coat. Technol..

[45] Yao Z., Liu M., Hu H., Tian J., Xu G. (2021). Microstructure and Wear Properties of a Bainite/Martensite Multi-phase Wear Resistant Steel. ISIJ Int..

